# Activation of photocatalytic CO_2_ reduction by loading hydrophobic thiolate-protected Au_25_ nanocluster cocatalyst†

**DOI:** 10.1039/d4na01045k

**Published:** 2025-02-12

**Authors:** Yuki Yamazaki, Yuki Tomoyasu, Tokuhisa Kawawaki, Yuichi Negishi

**Affiliations:** a Department of Applied Chemistry, Faculty of Science, Tokyo University of Science 1-3 Kagurazaka, Shinjuku-ku Tokyo 162-8601 Japan; b Carbon Value Research Center, Research Institute for Science & Technology, Tokyo University of Science 2641 Yamazaki Noda Chiba 278-8510 Japan; c Institute of Multidisciplinary Research for Advanced Materials, Tohoku University Katahira 2-1-1, Aobaku Sendai 980-8577 Japan

## Abstract

The photocatalytic carbon dioxide (CO_2_) reduction reaction (CO_2_RR), which reduces CO_2_ to various useful chemical compounds by light, has attracted attention to achieve carbon neutrality. In photocatalytic CO_2_RR, it is effective to load metal nanoparticles (NP) as cocatalysts on the surface of semiconductor photocatalysts to improve their activity and selectivity. In this study, we used ultrafine metal nanoclusters (NC) with a particle size of about 1 nm as cocatalysts to clarify the effect of surface ligands on the activity and selectivity of the photocatalytic CO_2_RR. As a result, it was shown that the introduction of hydrophobic ligands to the Au_25_ NC cocatalyst suppresses the competing hydrogen evolution reaction, thereby increasing the selectivity of CO_2_RR. In addition, the hydrophobic ligand-protected Au_25_ NC cocatalysts exhibited 66 times higher CO evolution rates per Au-loading weights than the Au NP cocatalysts with a particle size of about 7 nm. These results provide crucial insights into the creation of highly active metal NC cocatalysts for photocatalytic CO_2_RR.

## Abstract 2

A Au_25_ nanocluster cocatalyst with hydrophobic ligands significantly improves the photocatalytic CO_2_ reduction reaction activity with suppression of the competing H_2_ evolution reaction.

## Graphical abstract

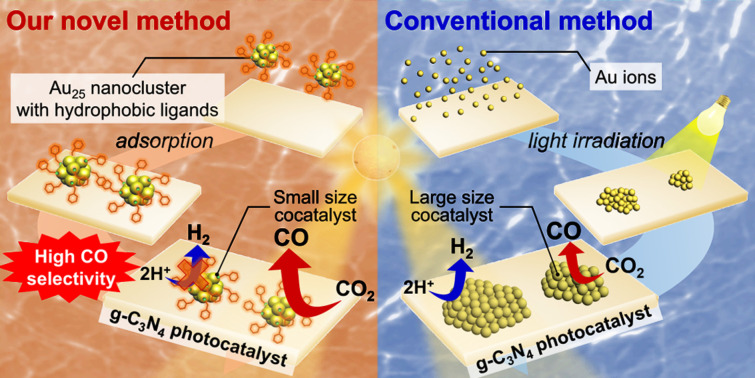

## Introduction

The photocatalytic carbon dioxide (CO_2_) reduction reaction (CO_2_RR), which reduces CO_2_ by light, is expected to be put to practical use as one of the means to solve energy, environmental and resource issues.^1,2^ This is because if exceedingly stable CO_2_ can be converted into various useful chemical compounds using sunlight, CO_2_ can be recycled and we can break free from the conventional energy systems that rely on fossil resources. For the practical use of the photocatalytic CO_2_RR, it is essential to further improve the efficiency and selectivity of the reduction products. Herein, commonly used photocatalysts are composed of a semiconductor photocatalyst and a metal/metal oxide cocatalyst. Importantly, many semiconductor photocatalysts that have a band gap suitable for CO_2_RR have been developed, and visible-light-driven photocatalysts that can respond to visible light rather than only ultraviolet light have been actively investigated in recent years.^3–7^ At the same time, the selection/fabrication of appropriate cocatalyst nanoparticles (NP) that have a significant impact on their activity and selectivity have also been developed. For example, it has been reported that Ag and Au NP cocatalysts mainly produce CO,^8–13^ whereas Pd, Cu and Rh–Ru alloy NP cocatalysts mainly produce CH_4_.^2,14–17^

**Fig. 1:**
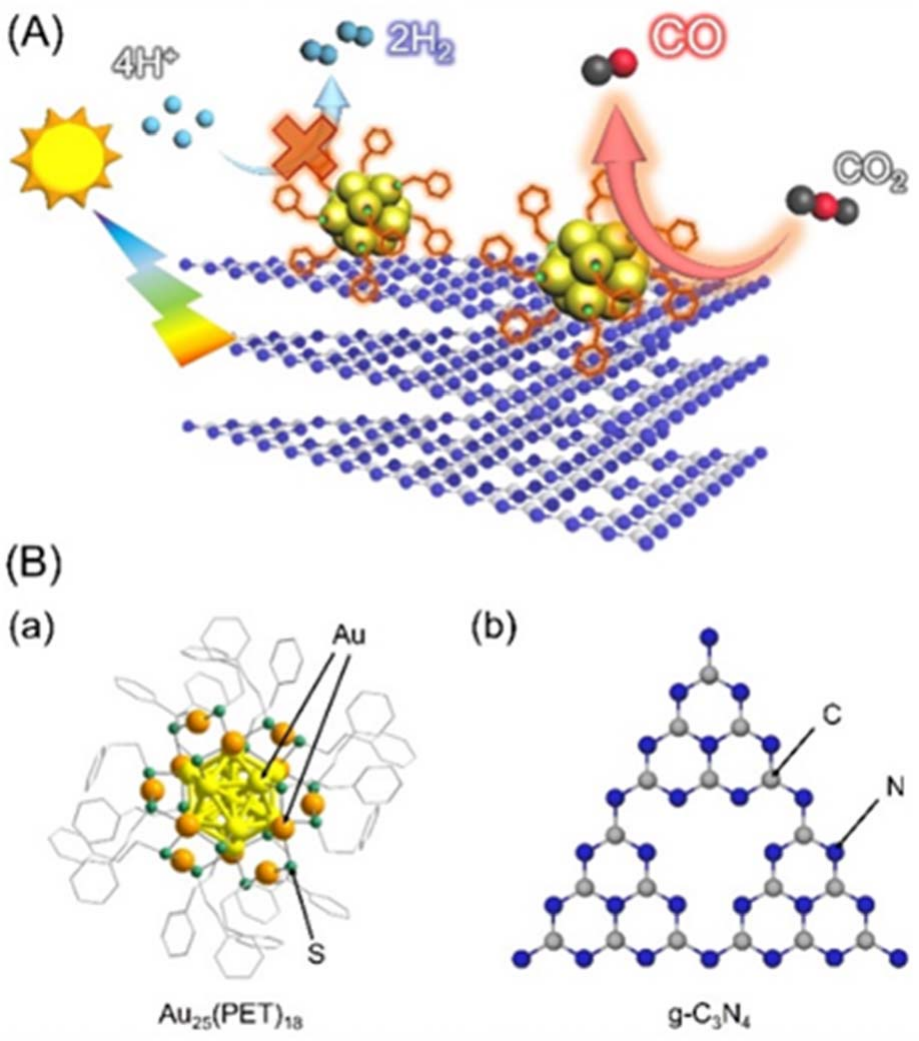
(A) Schematic of the purposes of this study and (B) geometric structure of (a) Au_25_(PET)_18_ and (b) gCN photocatalysts.

In addition, it is known that using smaller cocatalysts improves the activity of the catalytic reaction. In particular, when metal nanoclusters^18–25^ (NC) with a particle size of about 1 nm are used as cocatalysts, their photocatalytic activity for water splitting is greatly improved.^26–32^ This improvement is mainly due to (1) the increase in the number of active sites due to the increase in the specific surface area and (2) the optimization of the adsorption characteristics with the reaction substrates due to the change in the electronic state based on the quantum size effect. In general, the water splitting activity of metal NC cocatalysts for photocatalysts is improved when (i) their surfaces are protected by ligands with hydrophilic functional groups^33^ or (ii) the hydrophobic ligands are removed.^28,34^ This is because the presence of hydrophilic functional groups or removal of hydrophobic ligands makes it easier to be approached by protons (H^+^), which are the reaction substrate, on the surface metal atoms of the metal NC. By contrast, in photocatalytic CO_2_RR, the hydrogen evolution reaction (HER) due to water splitting is a competitive reaction. Therefore, if such photocatalytic HER can be suppressed by enhancing the hydrophobicity of the surface ligands, high selectivity and activity for photocatalytic CO_2_RR is expected to be obtained (Fig. 1A).

In this study, we attempted to experimentally verify such an assumption regarding the effects of the functionality of ligands of metal NC on photocatalytic CO_2_RR activity. Specifically, we used Au_25_ NC^35,36^ (Fig. 1B(a)), which are the most common model NC with high electrocatalytic CO_2_RR activity^37–40^ as cocatalysts and attempted to clarify the effects of hydrophobic and hydrophilic ligands that protect them during photocatalytic CO_2_RR activity. As a result, it was shown that the introduction of hydrophobic ligand-protected Au_25_ NC cocatalyst suppressed the competing HER, thereby increasing the selectivity of the CO_2_RR and it exhibited 66 times higher CO evolution rates per Au-loading weight than the Au NP cocatalysts made by a conventional photodeposition (PD) method.

## Results and discussion

In this study, we used g-C_3_N_4_ photocatalysts (gCN; Fig. 1B(b)), which have attracted much attention as a next-generation visible-light-driven photocatalyst because of their ease of synthesis, nontoxic nature, abundant availability of the raw materials on the Earth, and high physical and chemical stability.^41–45^ There have also been many reports of gCN as a photocatalyst for photocatalytic CO_2_RR.^46–48^ We synthesized gCN from urea by thermal polymerization as previously reported,^29^ and measured the powder X-ray diffraction and diffuse reflectance (DR) spectrum of the obtained gCN. From these results, we confirmed that the synthesized photocatalyst was visible-light-responsive g-C_3_N_4_ with relatively high crystallinity (Fig. S1†).

**Fig. 2:**
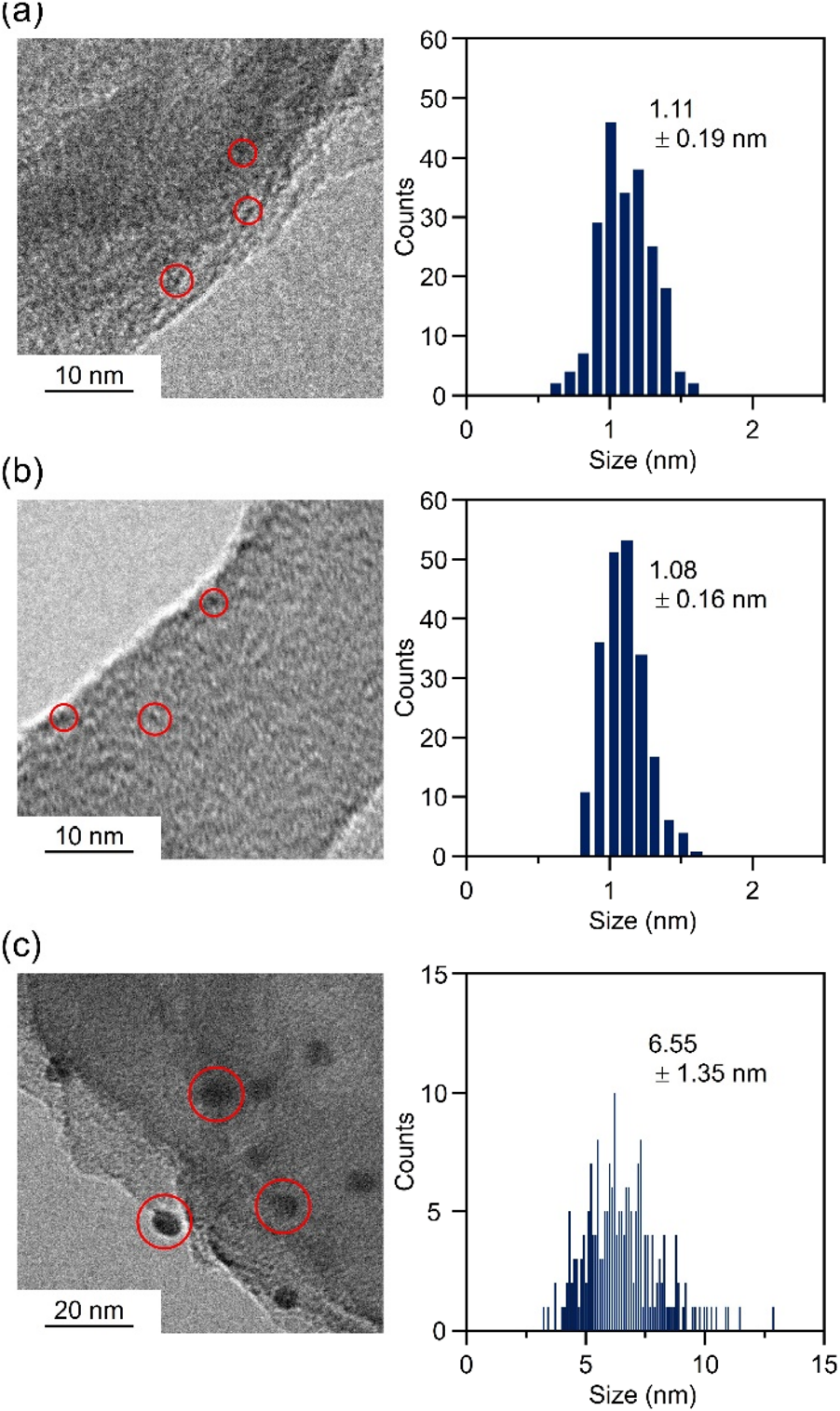
TEM images and resulting histograms of the particle-size distribution for Au cocatalysts for (a) Au_25_(PET)_18_/gCN, (b) Au_25_(PET, *p*-MBA)_18_/gCN and (c) Au NP/gCN. Loading amounts of Au are 0.1, 0.1 and 3.0 wt% for Au_25_(PET)_18_/gCN, Au_25_(PET, *p*-MBA)_18_/gCN and Au NP/gCN, respectively.

**Fig. 3:**
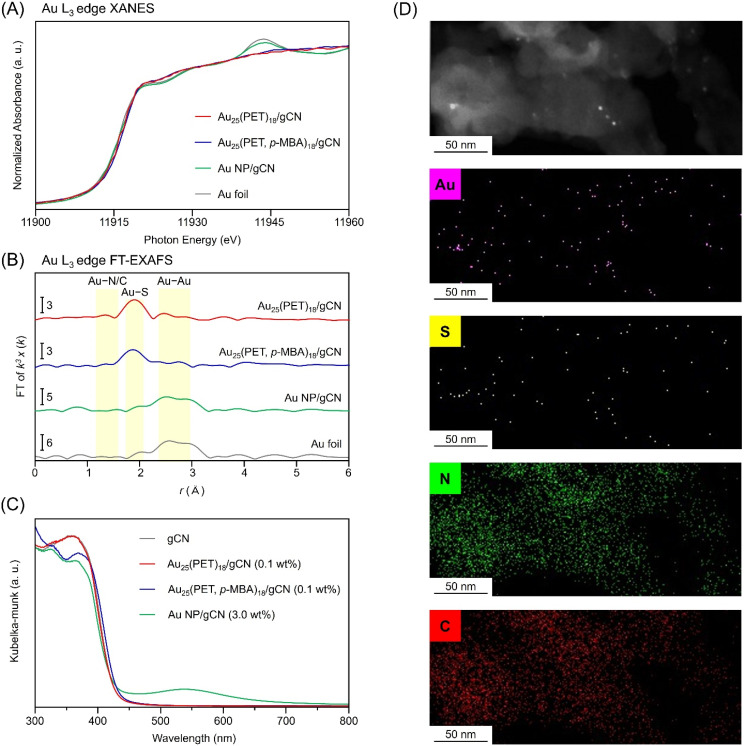
Characterization of cocatalyst-loaded gCN photocatalysts. Au L_3_-edge (A) XANES, (B) FT-EXAFS spectra and (C) DR spectra. (D) HAADF–STEM images and EDS elemental mapping of Au_25_(PET)_18_/gCN (Au–M, S–K, C–K and N–K). In (A and B), Au L_3_-edge XANES and FT-EXAFS spectra of Au foil is also shown for comparison. In (B), the peaks at ≈1.9 and 2.6–3.0 Å are assigned to the Au–S and Au–Au bonds, respectively. Loading amounts of Au are 0.1, 0.1 and 3.0 wt% for Au_25_(PET)_18_/gCN, Au_25_(PET, *p*-MBA)_18_/gCN and Au NP/gCN, respectively.

Next, Au_25_ NC protected by 2-phenylethanethiolate (PET), a hydrophobic ligand, was synthesized by a previously reported method (Au_25_(PET)_18_; Fig. S2 and S3†).^27,49^ The obtained Au_25_(PET)_18_ was dissolved in acetone and loaded on gCN using an impregnation method (Au_25_(PET)_18_/gCN). We characterized the prepared Au_25_(PET)_18_/gCN using various methods (Fig. 2 and S4†). The transmission electron microscopy (TEM) image of Au_25_(PET)_18_/gCN shown in Fig. 2a showed that the particle size of Au_25_(PET)_18_ cocatalysts on gCN (particle size: 1.11 ± 0.19 nm) was almost the same as Au_25_(PET)_18_ before adsorption on gCN (particle size: 1.03 ± 0.13 nm). Compared with the sizes of the Au NP cocatalysts of Au NP-loaded gCN (Au NP/gCN; particle size: 6.55 ± 1.35 nm; Fig. 2c and S5†) prepared using the conventional PD method, Au_25_(PET)_18_ cocatalysts have smaller and monodispersed sizes. The Au L_3_-edge X-ray absorption near edge structure (XANES) spectra showed that (1) the Au_25_(PET)_18_ cocatalyst on gCN has a metallic electronic state and (2) the electronic state of Au_25_(PET)_18_ is maintained before and after adsorption on gCN (Fig. 3A and S4†). Such the change in the electronic state between the Au cocatalyst and gCN was not observed by XPS (Fig. S6†). The Au L_3_-edge Fourier transform extended X-ray absorption fine structure (FT-EXAFS) spectra showed a peak (≈1.9 Å) due to the Au–S bond, suggesting that the SR ligands of Au_25_(PET)_18_ remain even after adsorption and that their geometric structure is mostly maintained (Fig. 3B and S4†). In the DR spectrum, there is no strong absorption in the visible region due to localized surface plasmon resonance, as seen in Au NP formed by aggregation (Fig. 3C and S4†). In an energy dispersive X-ray spectroscopy (EDS) elemental mapping obtained using a high-angle annular dark field–scanning TEM (HAADF–STEM) (Fig. 3D), Au and S were mapped to the same location, confirming that Au_25_(PET)_18_ cocatalyst was located on gCN. These analyses strongly indicated that Au_25_(PET)_18_ cocatalyst was loaded on gCN while largely maintaining its geometric structure.

**Fig. 4:**
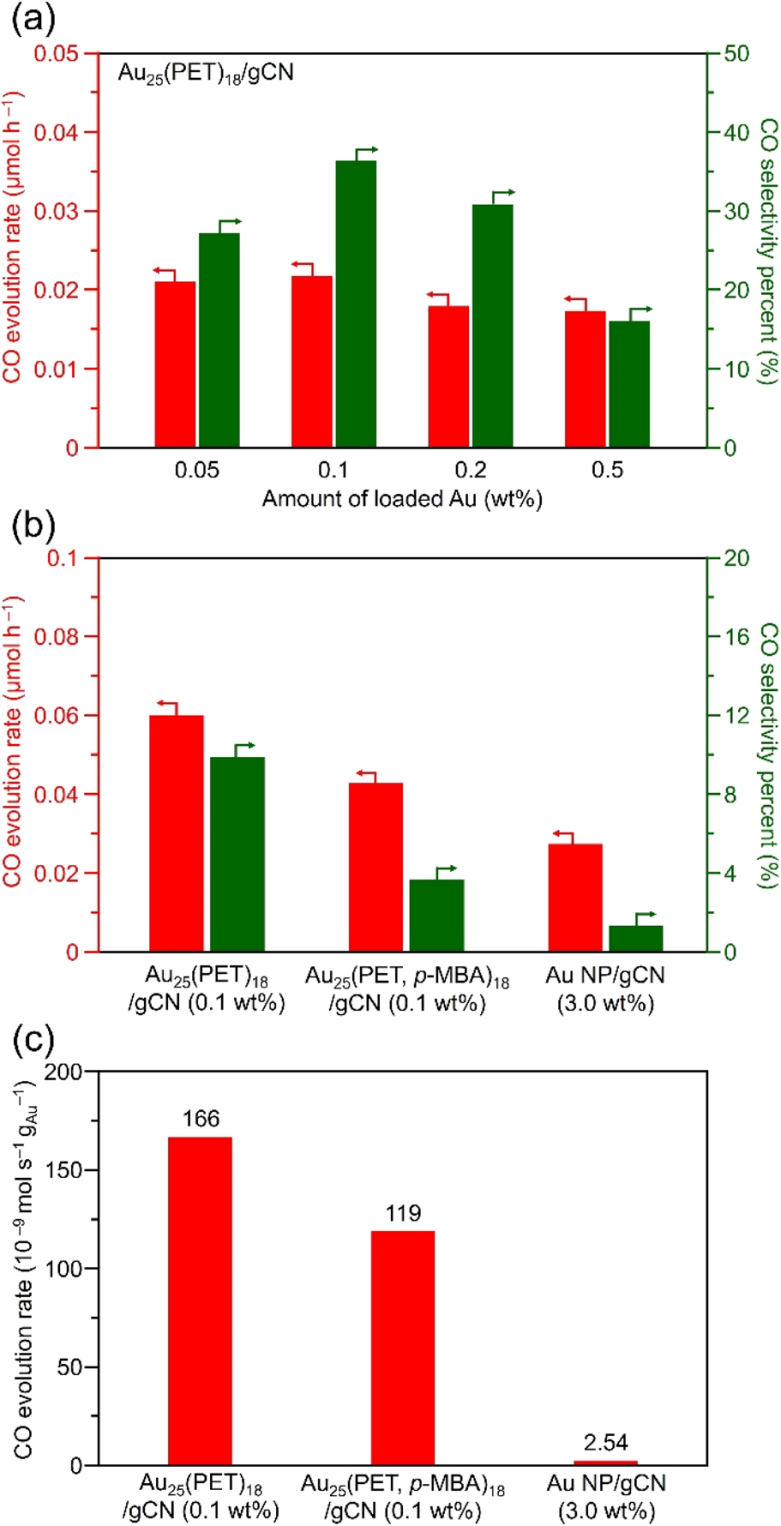
Photocatalytic CO_2_RR activity of Au cocatalyst-loaded gCN photocatalysts. (a) Dependence on loading weight of Au for Au_25_(PET)_18_/gCN. (b) Comparisons of CO evolution rates and resulting CO selectivities and (c) CO evolution rates per loading weight of Au, for Au_25_(PET)_18_/gCN, Au_25_(PET, *p*-MBA)_18_/gCN and Au NP/gCN. Photocatalyst: 100 mg, solution: water (60 mL) with TIPA (12.5 g), flow gas: CO_2_ (1 atm), light source: 405 nm LED lamp and cell: top-irradiation cell. In (b) and (c), loading weights of Au are 0.1, 0.1 and 3.0 wt% for Au_25_(PET)_18_/gCN, Au_25_(PET, *p*-MBA)_18_/gCN and Au NP/gCN, respectively.

The photocatalytic CO_2_RR activity of the Au_25_(PET)_18_/gCN was evaluated using a reaction cell under CO_2_ flow with light irradiation (Fig. 4). In the measurement, triisopropanolamine (TIPA) was added (20 vol%) as a hole sacrificial reagent to correctly evaluate the photocatalytic CO_2_RR activity.^50^ The Au cocatalyst-loaded gCN was dispersed in water with the sacrificial agent, it was irradiated with visible light using a blue LED (405 nm), and H_2_ or CO evolution was quantified using a gas chromatograph at regular time intervals (Fig. S7†). By analysing the reaction solution with a high-performance liquid chromatograph, it was confirmed that the product did not contain any liquid-phase CO_2_RR products. In addition, by evaluating the same measurement under Ar flow, it was confirmed that the obtained CO was derived from the flowing CO_2_ (Fig. S8†).

For the photocatalytic CO_2_RR experiments, we first investigated the optimal Au-loading weights of Au_25_(PET)_18_/gCN. As a result, the highest CO evolution rate was obtained when the Au-loading weight was set to 0.1 wt% (Fig. 4a). Therefore, in the subsequent experiments, we used Au_25_(PET)_18_/gCN prepared with this optimal 0.1 wt% of Au-loading weight.

Next, we investigated the effects of hydrophilic and hydrophobic ligands on Au_25_ NC on the photocatalytic CO_2_RR activity. In our previous report, we have succeeded in improving the hydrophilicity of Au_25_ NC by exchanging some of the ligands of Au_25_(PET)_18_ with 4-mercaptobenzoic acid (*p*-MBA), a hydrophilic ligand with a structure relatively similar to that of PET.^27^ Accordingly, we prepared Au_25_ NC in which some of the ligands were exchanged by *p*-MBA (Au_25_(PET, *p*-MBA)_18_; Fig. S2 and S3†), and loaded it on gCN by a liquid-phase adsorption method to prepare a photocatalyst (Au_25_(PET, *p*-MBA)_18_/gCN; Fig. 2b, 3 and S9†). The photocatalytic CO_2_RR activity of the obtained Au_25_(PET, *p*-MBA)_18_/gCN is shown in Fig. 4b. Interestingly, it was found that hydrophilic Au_25_(PET, *p*-MBA)_18_/gCN has 2.70 times lower CO selectivity (selectivity_CO_ = 3.66%) than hydrophobic Au_25_(PET)_18_/gCN (selectivity_CO_ = 9.87%). Jiang and Lee *et al.* reported that Au_25_(SR)_18_ protected by a hydrophilic ligand (3-mercaptopropanoic acid or 3-mercapto-1-propanesulfonic acid), which allowed efficient H^+^ relay, had a higher electrochemical HER than hydrophobic 1-hexanethiolate (SC_6_) ligand-protected Au_25_(SC_6_)_18_.^33^ In our study, the photocatalytic HER selectivity was also improved when hydrophilic Au_25_(PET, *p*-MBA)_18_/gCN was used, probably because *p*-MBA induced an effective H^+^ relay. Although hydrophilic NC were used as cocatalysts in photocatalytic CO_2_RR in the previous report,^51^ this study demonstrated that the use of these hydrophilic NC as cocatalysts (1) promoted the HER as a competing reaction and (2) thereby reduced the selectivity of the CO_2_RR (Fig. 4b and c).

Next, we compared the photocatalytic CO_2_RR activity between Au_25_(PET)_18_/gCN, which has a hydrophobic ligand suitable for photocatalytic CO_2_RR, and Au NP/gCN prepared using a conventional PD method (Fig. 4b). In this experiment, we also optimized the Au-loading weights of Au NP/gCN, which was found to be 3.0 wt% of Au (Fig. S10 and S11†). The results of the comparison for their photocatalytic CO_2_RR activity are also shown in Fig. 4b. The results demonstrated that (1) even though the Au-loading weight of Au_25_(PET)_18_/gCN was 30 times less than that of Au NP/gCN, Au_25_(PET)_18_/gCN shows higher CO evolution rate than Au NP/gCN (0.06 *vs.* 0.03 μmol_CO_ h^−1^, Fig. 4b) and (2) Au_25_(PET)_18_/gCN shows a greatly suppressed H_2_ evolution rate compared with Au NP/gCN (0.61 *vs.* 2.10 μmol_H2_ h^−1^, Fig. 4b). Comparing the CO evolution rate per Au-loading weight in Fig. 4c, Au_25_(PET)_18_/gCN and Au_25_(PET, *p*-MBA)_18_/gCN showed 66 and 47 times higher CO evolution rates than Au NP/gCN, respectively (166 *vs.* 119 *vs.* 2.54 × 10^−9^ mol s^−1^ g_Au_^−1^). Accordingly, Au_25_(PET)_18_/gCN showed the highest CO selectivity (9.87% *vs.* 3.66% *vs.* 1.31% for Au_25_(PET)_18_/gCN, Au_25_(PET, *p*-MBA)_18_ and Au NP/gCN, respectively). The previous study^38^ reported that Au_25_(SR)_18_ has a suitable CO_2_ adsorption site. Furthermore, molecular dynamics simulations suggested that the presence of hydrophilic ligands in [Au_25_(SR)_18_]^−^ efficiently induced HER due to enhanced proton transfer facilitated by hydrogen bonds.^52^ Therefore, it can be considered that Au_25_(PET)_18_/gCN promoted photocatalytic CO_2_RR with a relatively high CO selectivity because Au_25_(PET)_18_/gCN contains both the suitable CO_2_ adsorption site and hydrophobic PET which leads to the suppression of HER.

**Fig. 5:**
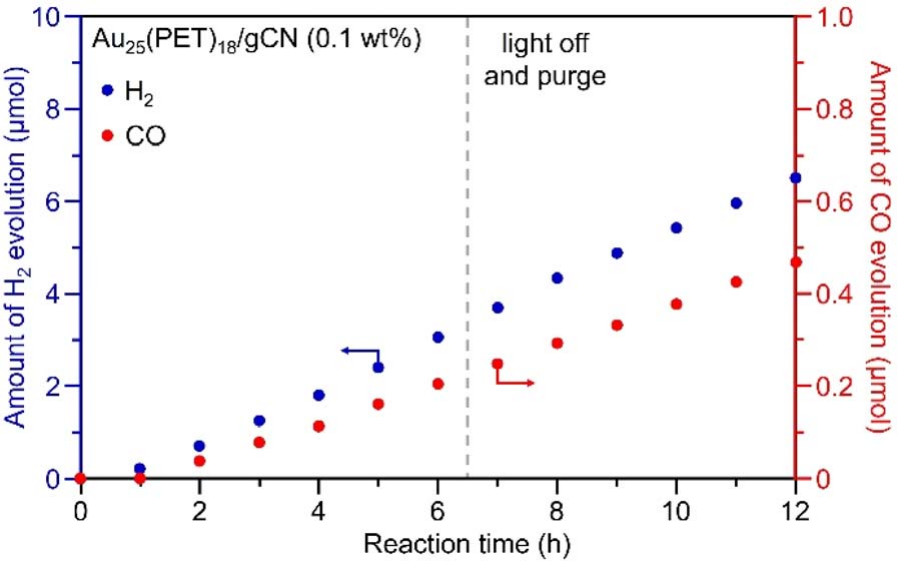
Time dependence of photocatalytic CO_2_RR activity of Au_25_(PET)_18_/gCN. Photocatalyst: 100 mg, solution: water (60 mL) with TIPA (12.5 g), flow gas: CO_2_ (1 atm), light source: 405 nm LED lamp and cell: top-irradiation cell. Loading weight of Au is 0.1 wt% for Au_25_(PET)_18_/gCN.

Finally, we evaluated the catalytic stability of Au_25_(PET)_18_/gCN, which has a relatively good CO evolution rate and selectivity. In general, the excited electrons generated by the photocatalytic reaction have a strong reduction power and may reductively decompose many organic substances. Therefore, we measured the long-term photocatalytic CO_2_RR activity of Au_25_(PET)_18_/gCN to evaluate its durability. As a result, it was shown that Au_25_(PET)_18_/gCN stably produced CO and its selectivity did not change even after 12 h of visible-light irradiation (Fig. 5). We also investigated the size of the cocatalyst after the photocatalytic activity test using TEM. As a result, the particle size of the Au_25_(PET)_18_ cocatalyst was almost the same as that of the Au_25_(PET)_18_ just after loading on gCN (particle size: 1.11 ± 0.17 nm; Fig. S4A†). In fact, no change in the diffraction pattern due to Au aggregation was observed in the powder X-ray diffraction (Fig. S12†). Au L_3_-edge XANES spectra showed that the electronic state of the Au_25_(PET)_18_ cocatalyst did not change significantly before and after light irradiation (Fig. S4C†). Furthermore, surprisingly, the peak (≈1.9 Å) derived from the Au–S bond was maintained in the Au L_3_-edge FT-EXAFS spectrum even after the evaluation of photocatalytic CO_2_RR activity (Fig. S4D†), suggesting that most of the ligands remain undecomposed even after long-term light irradiation. These results revealed that Au_25_(PET)_18_/gCN, which has a strong Au–S bond, has high stability against light irradiation.

## Conclusions

The loading of metal NC cocatalysts leads to a significant improvement in photocatalytic activity. In this study, we investigated the effect of organic ligands protecting the surface of these metal NC cocatalysts on the activity and selectivity of photocatalytic CO_2_RR. The results demonstrated that (1) the introduction of hydrophilic ligands to the Au_25_ NC cocatalyst promotes the competing HER, whereas (2) the introduction of hydrophobic ligands to the Au_25_ NC cocatalyst suppresses the HER, leading to the progress of photocatalytic CO_2_RR with relatively high CO selectivity. In this way, although it has been generally believed that the presence of organic ligands on the metal NC surface reduces photocatalytic activity, this study revealed that the presence of hydrophobic ligands effectively suppresses the competing HER in photocatalytic CO_2_RR. Furthermore, it was found that the hydrophobic Au_25_(PET)_18_-loaded photocatalysts show a 66 times higher CO evolution rate per Au-loading weight and relatively high CO selectivity compared with Au NP-loaded photocatalysts. These results will provide clear design guidelines for achieving high-performance photocatalytic CO_2_RR by loading metal NC cocatalysts. In the future, it is expected that the obtained knowledge will be applied to a semiconductor photocatalyst suitable for photocatalytic CO_2_RR^17^ to create high-performance CO_2_RR photocatalysts with significantly improved selectivity and activity, which will contribute to practical use (Fig. S13†).

## Data availability

All data generated in this study are provided in the manuscript and ESI.†

## Author contributions

T. K. and Y. N. conceived the research and designed the experiments. Y. Y., Y. T., and T. K. performed the syntheses, characterization and measurements of the CO_2_RR activity. T. K. and Y. N. wrote the manuscript.

## Conflicts of interest

There are no conflicts to declare.

## Supplementary Material

NA-007-D4NA01045K-s001
