# Supplementary material for: Activation of photocatalytic CO2 reduction by loading hydrophobic thiolate-protected Au25 nanocluster cocatalyst
Source: Nanoscale Adv. 2025 Feb 12;7(6):1518–23. doi: 10.1039/d4na01045k (PMC11833898; doi:10.1039/d4na01045k)
Supplement: NA-007-D4NA01045K-s001 [file NA-007-D4NA01045K-s001.pdf]

Supplementary Information (SI) for Nanoscale Advances.
This journal is © The Royal Society of Chemistry 2025

Supporting Information

Activation of Photocatalytic CO_2 Reduction by Loading Hydrophobic Thiolate-Protected Au_{25} Nanocluster Cocatalyst

Author Information

Yuki Yamazaki[1,‡], Yuki Tomoyasu[1,‡], Tokuhisa Kawawaki[1,2,*], and Yuichi Negishi[2,3,*]

Affiliations

[1]Department of Applied Chemistry, Faculty of Science, Tokyo University of Science, 1-3 Kagurazaka, Shinjuku-ku, Tokyo 162−8601, Japan

[2]Research Institute for Science & Technology, Tokyo University of Science, 2641 Yamazaki, Noda, Chiba 278−8510, Japan.,

[3]Institute of Multidisciplinary Research for Advanced Materials, Tohoku University, Katahira 2-1-1, Aoba-ku, Sendai 980-8577, Japan

[‡]These authors contributed equally to this work.

[*]Corresponding author E-mail: kawawaki@rs.tus.ac.jp (T.K.), yuichi.negishi.a8@tohoku.ac.jp (Y.N.)

1 Chemicals

All chemicals were obtained commercially and used without further purification. Hydrogen tetrachloroaurate tetrahydrate ($HAuCl_4 \cdot 4H_2O$) was obtained from Tanaka Kikinzoku. Tetraoctylammonium bromide (TOABr), Urea, sodium hydroxide (NaOH) bismuth standard solution (100 ppm), and gold standard solution (1000 ppm) were obtained from FUJIFILM Wako Pure Chemical Co.. Tetrahydrofuran (THF), methanol, toluene, acetone, hydrochloric acid (HCl), and nitric acid were obtained from Kanto Chemical Co., Inc. 4-Mercaptobenzoic acid (*p*-MBA) and 2-phenylethanethiol (PET) were obtained from Aldrich. Sodium tetrahydroborate ($NaBH_4$), Triisopropanolamine (TIPA; >98.0%; mixture of isomer), and trans-2-[3-(4-tert-Butylphenyl)-2-methyl-2-propenylidene]malononitrile (DCTB) were obtained from Tokyo Chemical Industry. Co., LTD. Pure Milli-Q water (>18.2 MΩ × cm) was generated with a Merck Millipore Direct 3 UV system.

2 Synthesis and Preparation

2.1 Synthesis of $Au_{25}(PET)_{18}$[1]

$Au_{25}(PET)_{18}$ was synthesized by the method reported by Jin and co-workers[2] with slight modification. First, 0.75 mmol of $HAuCl_4 \cdot 4H_2O$ was dissolved in 25 mL of a THF solution containing 0.76 mmol of TOABr. After the reaction mixture was stirred for 15 min, 4.7 mmol of PETwas added to the solution. After 30 min of stirring, 10 mL of a cold (0 °C) aqueous solution containing 8.7 mmol of $NaBH_4$ was rapidly added. After 10 h of stirring, THF was evaporated, and the remaining red-brown powder was washed three times with a mixture of methanol and water (4:1), followed by washing with methanol to remove excess thiol and other byproducts. Finally, pure $Au_{25}(PET)_{18}$ was extracted from the precipitate of the mixture using toluene.

2.2. Synthesis of Au_{25}(PET, *p*-MBA)$_{18}$[3]

$Au_{25}(PET)_x(p\text{-MBA})_{18-x}$ (Au_{25}(PET, *p*-MBA)$_{18}$) was prepared by exchanging a part of the ligand of $Au_{25}(PET)_{18}$ with *p*-MBA (not thiol but thiolate). In the experiment, first, 3 mg of $Au_{25}(PET)_{18}$ was dissolved in 1 mL of acetone. Then, 3 mg of *p*-MBAwas added to this solution. The solution was left at room temperature for 2 h. The obtained product was dried by evaporation and then the dried product was washed with a mixture of methanol and water (7:3) to remove excess thiol and other byproducts. This washing operation was repeated at least five times. The obtained Au_{25}(PET, *p*-MBA)$_{18}$ with a lower number of *p*-MBA ligands was then reacted again with *p*-MBAunder the same experimental conditions to increase the number of *p*-MBA ligands in Au_{25}(PET, *p*-MBA)$_{18}$.

2.3. Synthesis of gCN[4]

In accordance with previous studies,[5,6] g-C_3N_4 (gCN) was synthesized by thermal polymerization of urea. First, approximately 10 g of urea was placed in an alumina crucible with a lid. The crucible was then placed in a muffle oven, and the temperature was increased to 550 °C at 5 °C min^{-1} in air, and heating was continued for 2 h. A light-yellow powder was obtained by cooling the product to 25 °C. The obtained powder was washed with ultrapure water, 1 M HCl, and 1 M NaOH, and it was then washed three times with ultrapure water to remove all of the unreacted and possibly harmful surface species. Finally, it was dried under reduced pressure to obtain a yellow powder (gCN).

2.4. Preparation of $Au_{25}(PET)_{18}$/gCN

$Au_{25}(PET)_{18}$/gCN, where $Au_{25}(PET)_{18}$ were loaded on gCN by the impregnation method. gCN (300 mg) and acetone (5 mL) were added to an agate mortar and dispersed by stirring with an agate pestle for 15 min. The objective loading amount of $Au_{25}(PET)_{18}$ was added, and acetone was evaporated while stirring in the same manner. Finally, it was dried under reduced pressure to obtain $Au_{25}(PET)_{18}$/gCN.

2.5. Preparation of Au_{25}(PET, *p*-MBA)$_{18}$/gCN

Au_{25}(PET, *p*-MBA)$_{18}$/gCN, where Au_{25}(PET, *p*-MBA)$_{18}$ were loaded on gCN by the liquid-phase adsorption method. In accordance with previous studies,[1] gCN (300 mg) and acetone (50 mL) were added to a Erlenmeyer flask and dispersed by ultrasonication for 15 min. Acetone solution containing the objective loading amount of Au_{25}(PET, *p*-MBA)$_{18}$ was added with vigorous stirring to obtain a total volume of 100 mL. After 1 h, the powder was collected by centrifugation and dried under reduced pressure. To determine the actual adsorbed amount of Au_{25}(PET, *p*-MBA)$_{18}$, the amount of Au contained in the supernatant solution was measured by inductively coupled plasma mass spectrometry (ICP-MS). Finally, it was dried under reduced pressure to obtain Au_{25}(PET, *p*-MBA)$_{18}$/gCN.

2.6. Preparation of Au NP/gCN[4]

Au NP/gCN loaded with Au nanoparticles (NP) by the photodeposition method was prepared based on a previous report.[5] gCN (300 mg) and ultrapure water (315 mL) were added to an internally irradiated reaction vessel and dispersed by ultrasonication for 15 min. While stirring the obtained suspension, the objective loading amount of $HAuCl_4 \cdot 4H_2O$ and 35 mL of methanol were added. After replacing the air in the solution with argon (Ar), the Ar flow rate was adjusted to 30 mL min^{-1} and the solution was irradiated with a high-pressure mercury lamp for 3 h. The powder was collected by centrifugation and dried. Finally, the obtained powder was photoactivated by visible-light irradiation to obtain Au NP/gCN.

3. Characterization

The powder X-ray diffraction (PXRD) patterns of the samples were measured with a Rint2500 diffractometer (Rigaku) using Cu-Kα source operated at 40 kV and 200 mA. A reflection-free silicon plate was used as a substrate.

The transmission electron microscope (TEM) images were recorded with a H-9500 electron microscope (HITACHI, Tokyo, Japan) or JEM-2100 electron microscope (JEOL, Tokyo, Japan) operating at 200 kV, typically using magnification of 500,000.

The high-angle annular dark field scanning TEM (HAADF-STEM) and annular bright field scanning TEM (ABF-STEM) were obtained by ultra-high-resolution transmission electron microscope (The Thermo Fisher Scientific Titan

G2 80−200) operating at 200 kV, with a beam convergence semi angle of 21.4 mrad, HAADF collection angle from 80−200 mrad and ABF collection angle from 11-22 mrad. Elemental maps were acquired using a super-X detector and low-background sample holder.

Au L_3-edge X-ray absorption fine structure (XAFS) measurements were performed at beamline BL01B1 of the SPring-8 facility of the Japan Synchrotron Radiation Research Institute (proposal numbers 2023B1825, 2024A1698). The incident X-ray beam was monochromatized by Si (111) for Au L_3 edge. XAFS spectra of Au foil as references and the samples were recorded in transmission mode using ionization chambers. The X-ray energy was calibrated for the Au L_3-edge using Au foil. The XANES and EXAFS spectra were analyzed using xTunes[7] as follows. The χ spectra were extracted by subtracting the atomic absorption background using cubic spline interpolation and normalized to the edge height. The normalized data were used as the XANES spectra. The k^3-weighted χ spectra in the k range of 3.0–12.0 $Å^{-1}$ were Fourier transformed into r space for structural analysis.

The ultraviolet-visible (UV-vis) absorption spectra of products were acquired in water solution at room temperature with a V-670 spectrometer (JASCO, Tokyo, Japan).

The diffuse reflection (DR) spectra were acquired at ambient temperature with a V-670 spectrometer (JASCO, Tokyo, Japan). The wavelength-dependent optical data [$I(w)$] were converted to energy-dependent data [I(E)] with the following equation that conserved the integrated spectral areas: $I(E) = I(w)/|\partial E/\partial w| \propto I(w) \times w^2$.

ICP-MS was performed with an Agilent 7850c spectrometer (Agilent Technologies, Tokyo, Japan). Bismuth was used as the internal standard. The ICP-MS measurements were performed for the supernatant obtained after adsorption $Au_{25}(PET)_{18}$ and $Au_{25}(PET, p\text{-MBA})_{18}$ with the photocatalyst to estimate the unabsorbed or loaded Au content.

A JMS-S3000 spiral time-of-flight mass spectrometer (JEOL, Tokyo, Japan) equipped with a semiconductor laser ($\lambda = 349$ nm) was used for measuring Matrix-assisted laser desorption/ionization (MALDI)-mass spectra (MS). DCTB was used as the MALDI matrix. To minimize NC dissociation induced by laser irradiation, DCTB was used as sample matrix with a sample-to matrix ratio of around 1:1000.

CO_2 adsorption was measured at 0 °C using Autosorb-iQ (Anton Paar, USA) in the pressure range 20-800 torr.

4. Measurements of photocatalytic activity

4.1 Photocatalytic CO_2 reduction reaction

Measurements of durability of photocatalytic CO_2 reduction reaction (CO_2RR) was performed at 25 °C using a flow system with a pyrex glass upper irradiation cell. The reaction was performed with an CO_2 gas flow rate of 15 mL min^{-1}. Before the measurements, the reaction solution containing the prepared photocatalysts (100 mg) and TIPA (12.5 g) as a hole sacrificial reagent in ultrapure water (60 mL) was purged with CO_2 gas for 1 h to ensure complete removal of air from the reaction vessel. The suspension was irradiated with a LED lamp (Asahi Spectra, CL-1501) with a LED head (Asahi Spectra, CL-H1-405-9-1-B), with an irradiation area was 32 cm^2 at 405 nm. The amounts of evolved gases were determined using online gas chromatographs (Shimadzu GC-8A equipped with the MS-5A column, a TCD, and an Ar carrier for H_2; and Shimadzu GC-8A equipped with the MS-13X column, a FID with methanizer, and an N_2 carrier for CO).

4.2 Durability measurement

Measurements of durability of photocatalytic CO_2RR was performed at 25 °C using a flow system with a pyrex glass upper irradiation cell. The reaction was performed with an CO_2 gas flow rate of 15 mL min^{-1}. Before the measurements, the reaction solution containing the prepared photocatalysts (100 mg) and TIPA (12.5 g) as a hole sacrificial reagent in ultrapure water (60 mL) was purged with CO_2 gas for 1 h to ensure complete removal of air from the reaction vessel. The suspension was irradiated with a LED lamp (Asahi Spectra, CL-1501) with a LED head (Asahi Spectra, CL-H1-405-9-1-B), with an irradiation area was 30 cm^2. The power of incident light at the center was adjusted to 82 mW cm^2 for 12 hours-test at 405 nm. The amounts of evolved gases were determined using online gas chromatographs (Shimadzu GC-8A equipped with the MS-5A column, a TCD, and an Ar carrier for a H_2 detection; and Shimadzu GC-8A equipped with the MS-13X column, a FID with methanizer, and an N_2 carrier for a CO detection).

5. Additional Figures

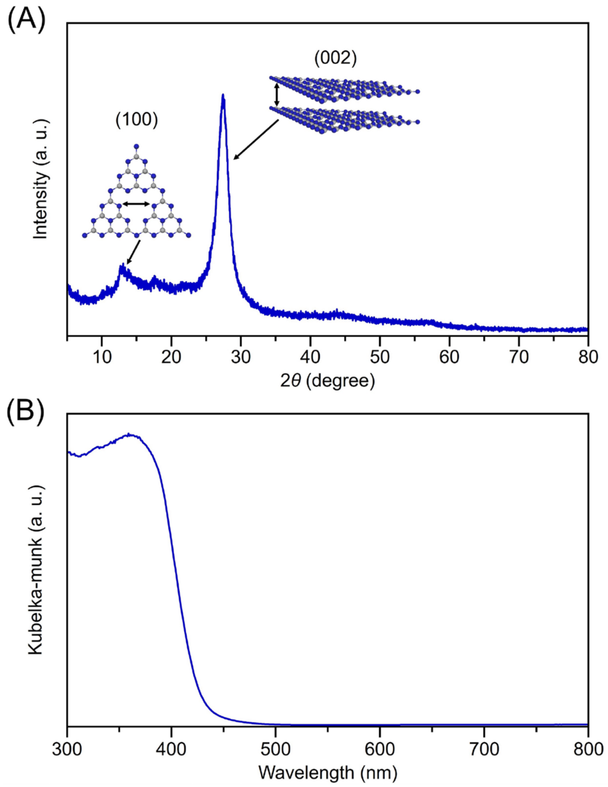

Figure S1. (A) PXRD patterns and (B) DR spectra of synthesized gCN.

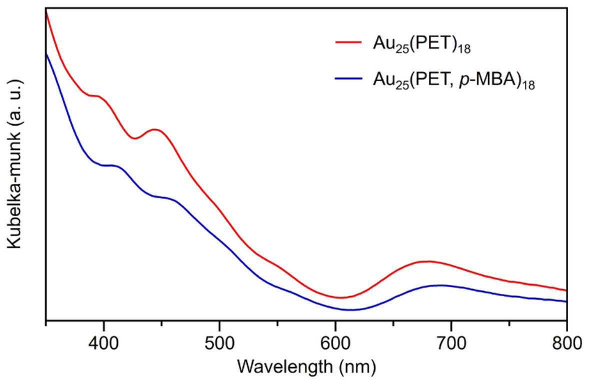

Figure S2. UV-vis spectra of $Au_{25}(PET)_{18}$ and $Au_{25}(PET, p\text{-MBA})_{18}$ in acetone solution, respectively.

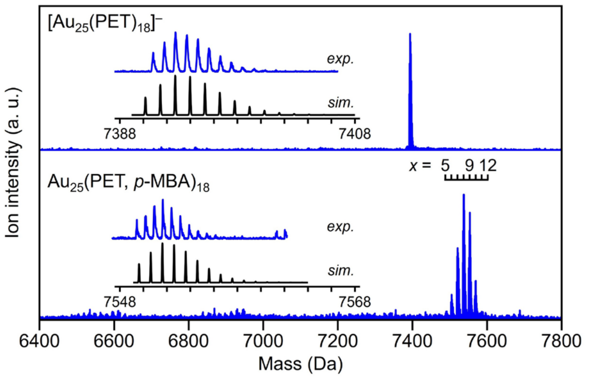

Figure S3. MALDI-MS spectra of $Au_{25}(PET)_{18}$ and $Au_{25}(PET, p\text{-MBA})_{18,}$ respectively.

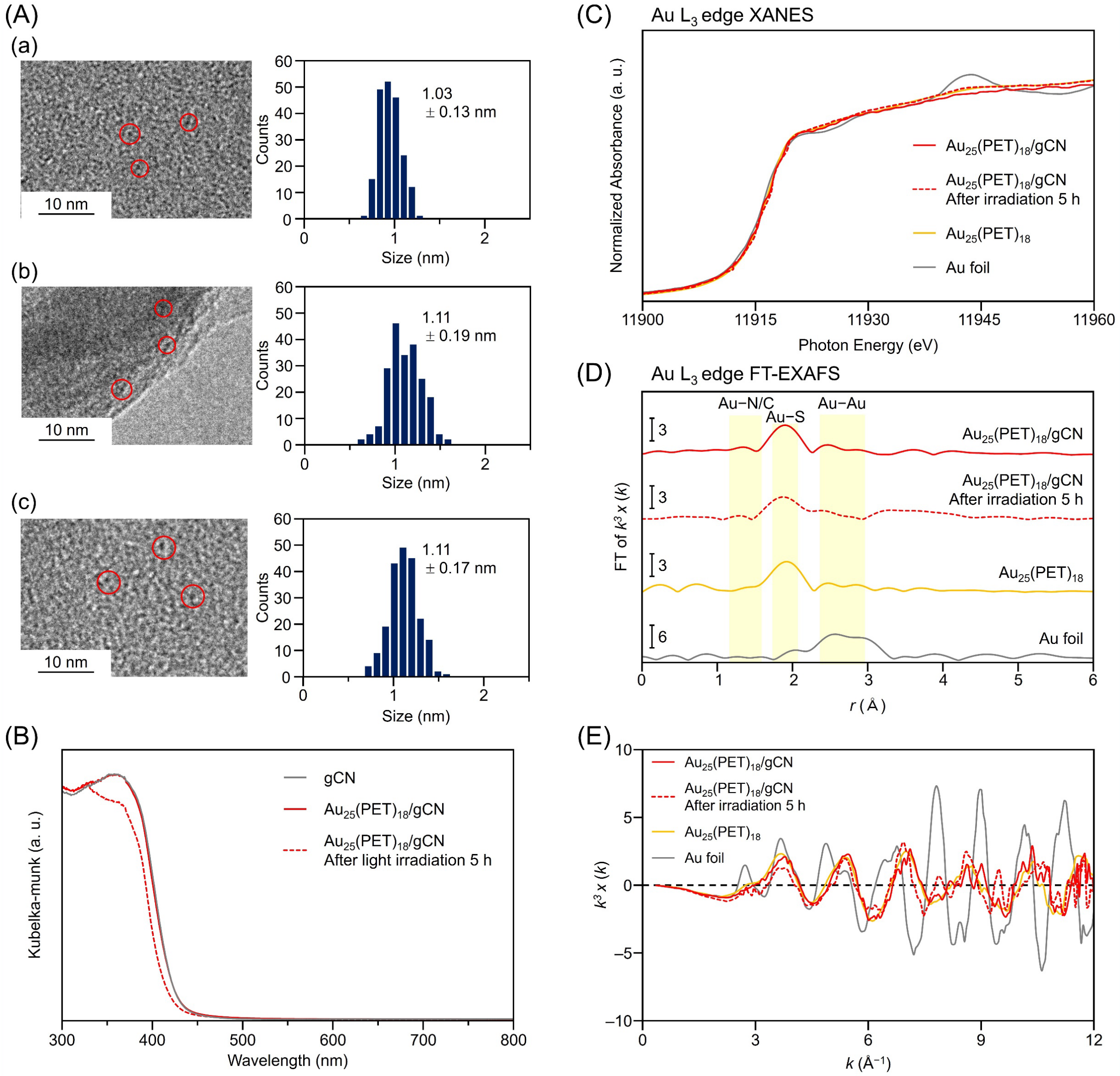

Figure S4. Characterization of $Au_{25}(PET)_{18}$/gCN. (A) TEM images (a) before and (b) after adsorption of $Au_{25}(PET)_{18}$ on gCN and (c) after photocatalytic reaction for 5 h of $Au_{25}(PET)_{18}$/gCN. (B) DR spectra and Au L_3-edge (C) XANES, (D) FT-EXAFS and (E) EXAFS spectra of before and after photocatalytic reaction for 5 h of $Au_{25}(PET)_{18}$/gCN. In (C–E), Au foil and $Au_{25}(PET)_{18}$ are also shown for comparison. In (D), the peak at ~1.9 and 2.6−3.0 Å are assigned to the Au–S, and Au–Au bond, respectively. In (A–E), loading amount of Au is 0.1 wt%.

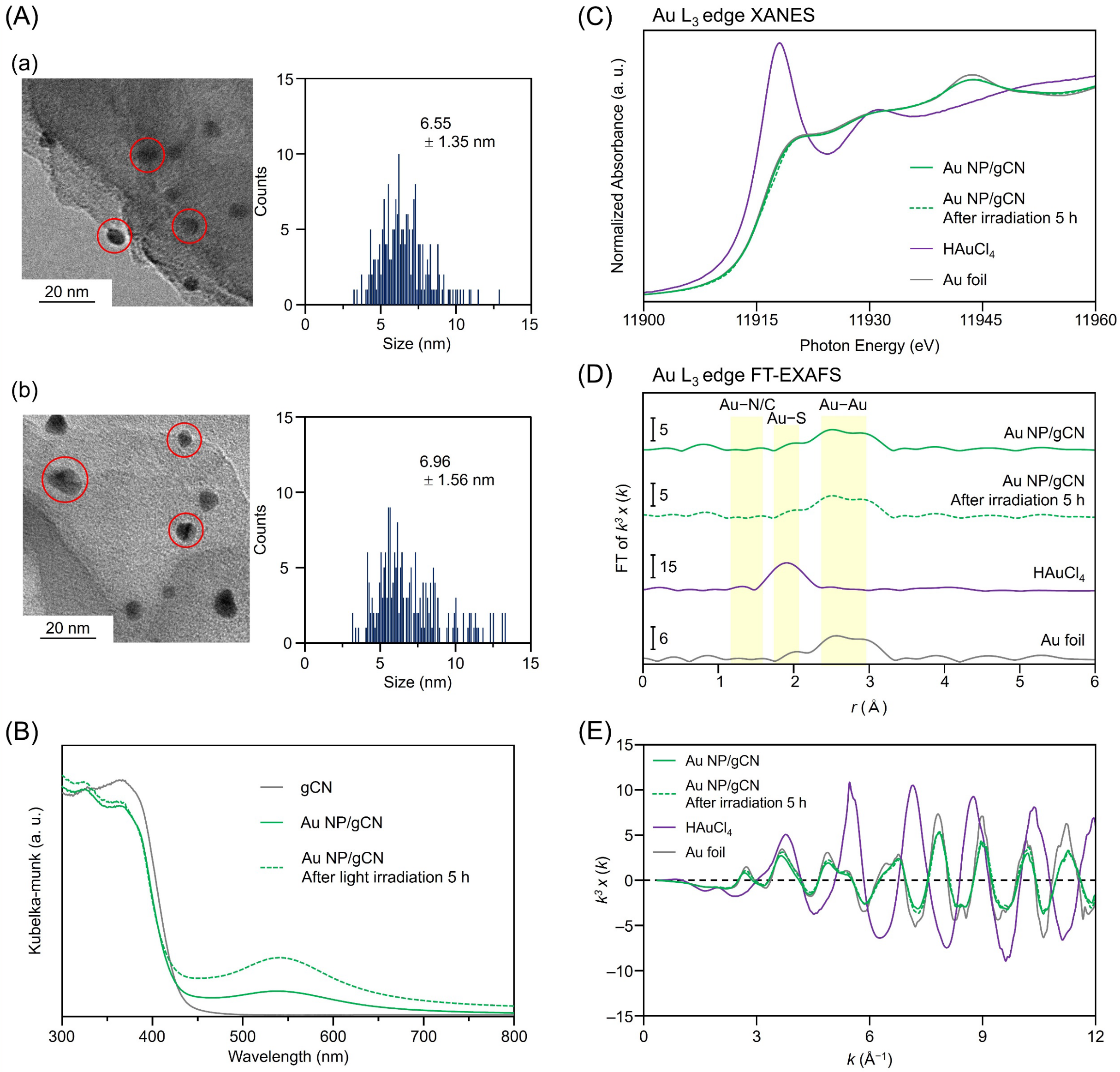

Figure S5. Characterization of Au NP/gCN. (A) TEM images (a) before and (b) after photocatalytic reaction for 5 h of Au NP/gCN. (B) DR spectra and Au L_3-edge (C) XANES, (D) FT-EXAFS and (E) EXAFS spectra of before and after photocatalytic reaction for 5 h of Au NP/gCN prepared by photodeposition method. In (C–E), Au foil and $HAuCl_4$ powder are also shown for comparison. In (D), the peak at ~1.9 and 2.6−3.0 Å are assigned to the Au–S, and Au–Au bond, respectively. In (A–E), loading amount of Au is 3.0 wt%.

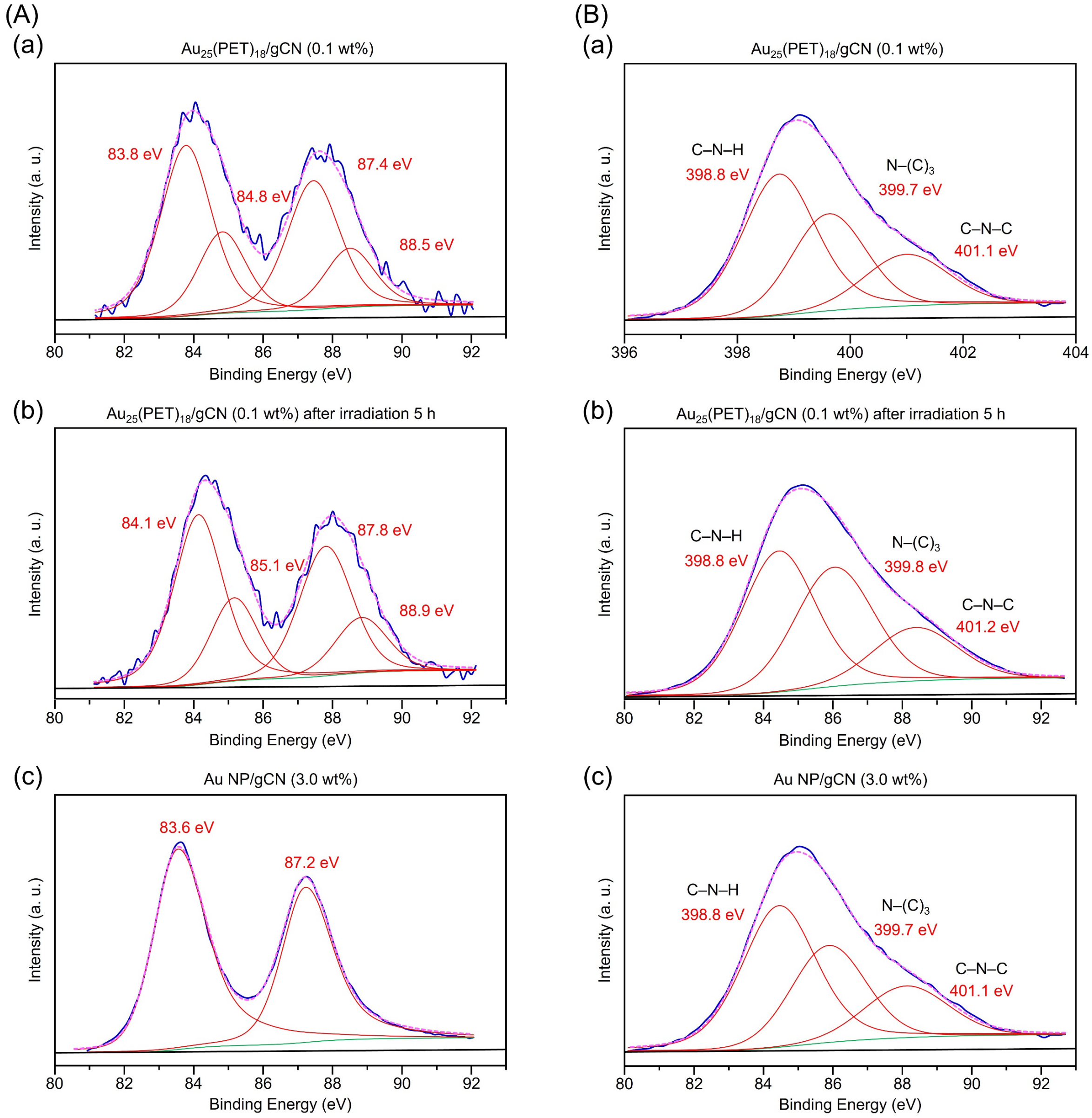

Figure S6. (A) Au 4f and (B) N 1s XPS spectrum of (a) before and (b) after photocatalytic CO_2RR for $Au_{25}(PET)_{18}$/gCN, and (c) Au NP/gCN.

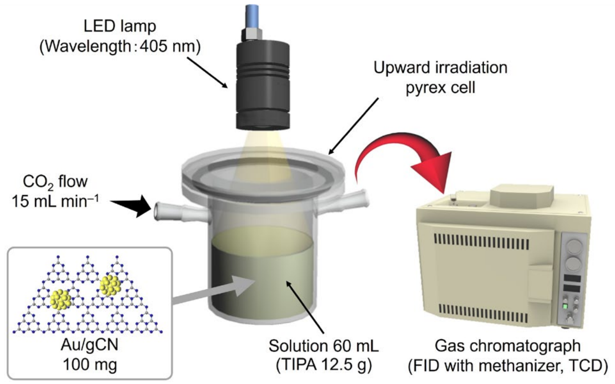

Figure S7. Schematic of the measurements system for photocatalytic CO_2 reduction.

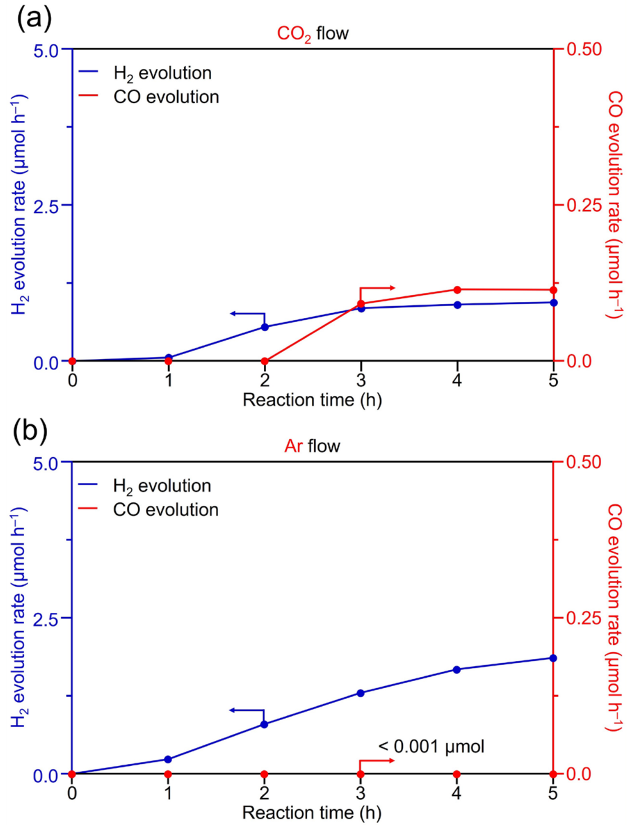

Figure S8. Results of photocatalytic reaction under (a) CO_2 or (b) Ar flow for $Au_{25}(PET)_{18}$/gCN (0.1 wt% of Au)

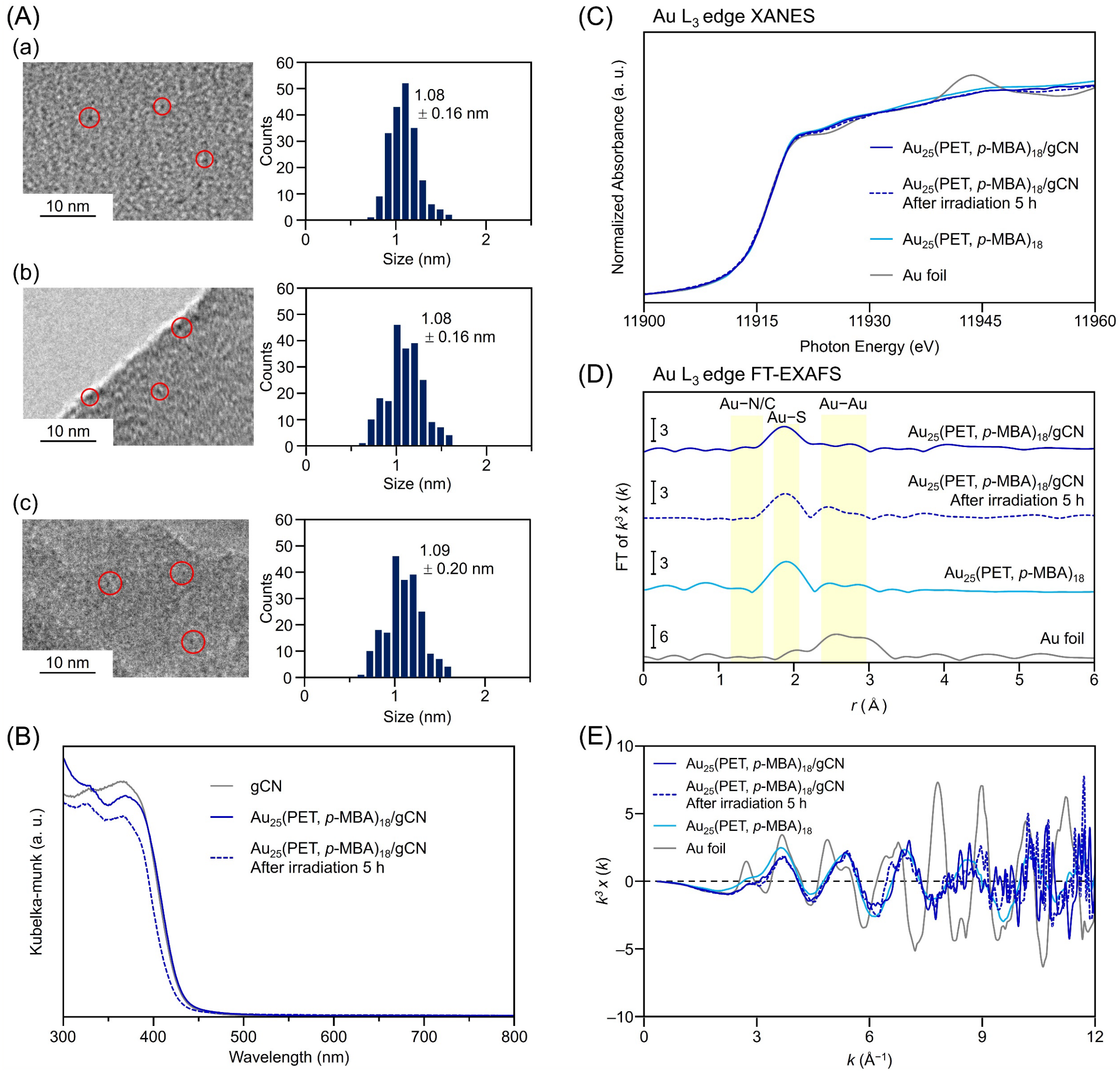

Figure S9. Characterization of Au_{25}(PET, *p*-MBA)$_{18}$/gCN. (A) TEM images (a) before and (b) after adsorption of Au_{25}(PET, *p*-MBA)$_{18}$ on gCN and (c) after photocatalytic reaction for 5 h of Au_{25}(PET, *p*-MBA)$_{18}$/gCN. (B) DR spectra and Au L_3-edge (C) XANES, (D) FT-EXAFS and (E) EXAFS spectra of before and after photocatalytic reaction for 5 h of Au_{25}(PET, *p*-MBA)$_{18}$/gCN. In (C–E), Au foil and Au_{25}(PET, *p*-MBA)$_{18}$ are also shown for comparison. In (D), the peak at ~1.9 and 2.6−3.0 Å are assigned to the Au–S, and Au–Au bond, respectively. In (A–E), loading amount of Au is 0.1 wt%.

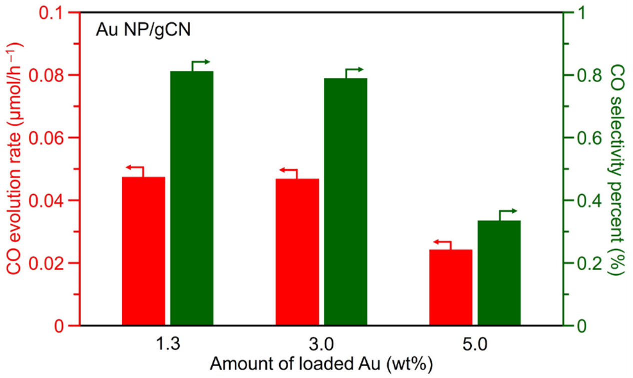

Figure S10. Photocatalytic CO_2RR activity of Au NP/gCN with different loading weight of Au from 1.3 to 3.0 wt%. Photocatalyst: 100 mg, solution: water (60 mL) with TIPA (12.5 g), flow gas: CO_2 (1 atm), light source: 405 nm LED lamp, and cell: top-irradiation cell. The highest CO evolution rate was achieved at 3.0 wt% of Au loading.

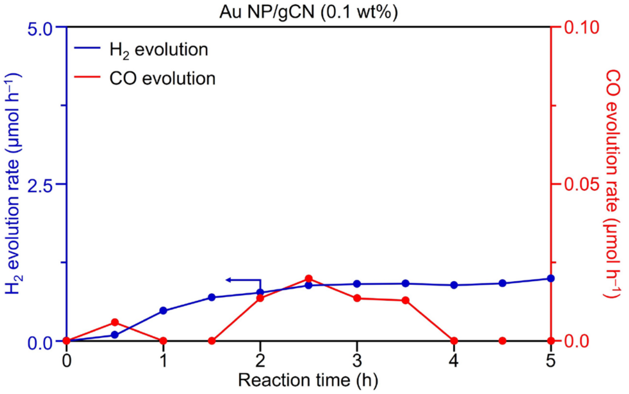

Figure S11. Time courses of photocatalytic CO_2RR activity of Au NP/gCN with 0.1 wt% of Au loading. Photocatalyst: 100 mg, solution: water (60 mL) with TIPA (12.5 g), flow gas: CO_2 (1 atm), light source: 405 nm LED lamp, and cell: top-irradiation cell. Au NP/gCN with a Au loading of 0.1 wt% showed almost no photocatalytic CO_2RR activity.

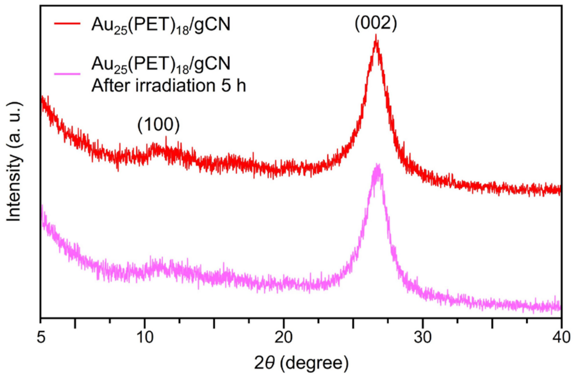

Figure S12. PXRD patterns of $Au_{25}(PET)_{18}$/gCN before and after photocatalytic reaction for 5 h.

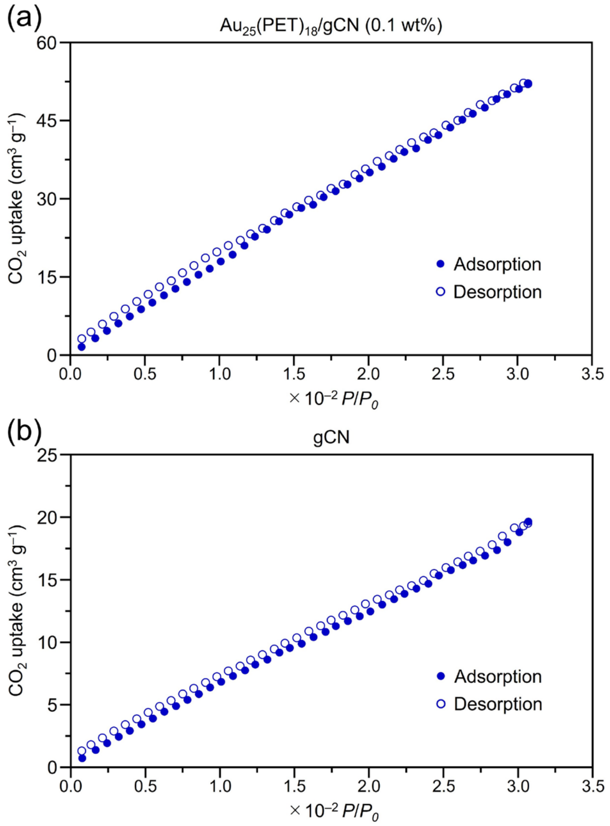

Figure S13. Results of CO_2 adsorption test for (a) $Au_{25}(PET)_{18}$/gCN and (b) gCN.
The g-C_3N_4 does not necessarily have high adsorption properties for CO_2. As a result, it is presumed that the photocatalytic CO_2RR activity was relatively low.

6. Reference

[1] T. Kawawaki, Y. Kataoka, M. Hirata, Y. Akinaga, R. Takahata, K. Wakamatsu, Y. Fujiki, M. Kataoka, S. Kikkawa, A. S. Alotabi, S. Hossain, D. J. Osborn, T. Teranishi, T. G. G.Andersson, G. F. Metha, S. Yamazoe and Y. Negishi, *Angew. Chem. Int. Ed.*, 2021, **60,** 21340–21350.

[2] H.Qian, C. Liu and R. Jin, *Sci. China:Chem.*, 2012, **55**, 2359–2365.

[3] W. Kurashige, R. Hayashi, K. Wakamatsu, Y. Kataoka, S. Hossain, A. Iwase, A. Kudo, S. Yamazoe and Y. Negishi, *ACS Appl. Energy Mater.*, 2019, **2**, 4175–4187.

[4] D. Yazaki, T. Kawawaki, D. Hirayama, M. Kawachi, K. Kato, S. Oguchi, Y. Yamaguchi, S. Kikkawa, Y. Ueki, S. Hossain, D. J. Osborn, F. Ozaki, S. Tanaka, J. Yoshinobu, G. F. Metha, S. Yamazoe, A. Kudo, A. Yamakata and Y. Negishi, *Small*, 2023, **19**, 2208287.

[5] G. Zhang, Z.-A. Lan, L. Lin, S. Lin and X. Wang, *Chem. Sci.*, 2016, **7**, 3062–3066.

[6] D. J. Martin, K. Qiu, S. A. Shevlin, A. D. Handoko, X. Chen, Z. Guo and J. Tang, *Angew. Chem., Int. Ed.*, 2014, **53**, 9240–9245.

[7] H. Asakura, S. Yamazoe, T. Misumi, A. Fujita, T. Tsukuda and T. Tanaka, *Radiat. Phys. Chem.*, 2020, **175**, 108270.
